# System dynamics models of depression at the population level: a scoping review

**DOI:** 10.1186/s12961-023-00995-7

**Published:** 2023-06-13

**Authors:** Eva Graham, Geneviève Gariépy, Heather Orpana

**Affiliations:** 1 Centre for Surveillance and Applied Research, Health Promotion and Chronic Disease Prevention Branch, Public Health Agency of Canada, 785 Carling Ave, Ottawa, ON Canada; 2 Department of Social and Preventive Medicine, School of Public Health, University of Montreal, Montreal, Canada; 3 Montreal Mental Health University Institute Research Center, Montreal, Canada; 4 School of Epidemiology and Public Health, University of Ottawa, Ottawa, Canada; 5 Dalla Lana School of Public Health, University of Toronto, Toronto, Canada

**Keywords:** Depression, System dynamics modelling, Simulation modelling, Scoping review

## Abstract

**Aims:**

Depression is a disease driven by dynamic processes both at the individual- and system-level. System dynamics (SD) models are a useful tool to capture this complexity, project the future prevalence of depression and understand the potential impact of interventions and policies. SD models have been used to model infectious and chronic disease, but rarely applied to mental health. This scoping review aimed to identify population-based SD models of depression and report on their modelling strategies and applications to policy and decision-making to inform research in this emergent field.

**Methods:**

We searched articles in MEDLINE, Embase, PsychInfo, Scopus, MedXriv, and abstracts from the System Dynamics Society from inception to October 20, 2021 for studies of population-level SD models of depression. We extracted data on model purpose, elements of SD models, results, and interventions, and assessed the quality of reporting.

**Results:**

We identified 1899 records and found four studies that met the inclusion criteria. Studies used SD models to assess various system-level processes and interventions, including the impact of antidepressant use on population-level depression in Canada; the impact of recall error on lifetime estimates of depression in the USA; smoking-related outcomes among adults with and without depression in the USA; and the impact of increasing depression incidence and counselling rates on depression in Zimbabwe. Studies included diverse stocks and flows for depression severity, recurrence, and remittance, but all models included flows for incidence and recurrence of depression. Feedback loops were also present in all models. Three studies provided sufficient information for replicability.

**Conclusions:**

The review highlights the usefulness of SD models to model the dynamics of population-level depression and inform policy and decision-making. These results can help guide future applications of SD models to depression at the population-level.

**Supplementary Information:**

The online version contains supplementary material available at 10.1186/s12961-023-00995-7.

## Background

Healthcare systems and policymaking face complex problems that involve the actions of multiple stakeholders, interventions, and processes working simultaneously. Many elements of the healthcare system are complex and include elements that are unpredictable, dynamic, and may change non-linearly, such as through tipping points [1, 2]. Traditional epidemiological and health systems research often fails to account for this complexity and properties of dynamic systems such as emergence, feedback, and adaptation [2, 3]. Emergence describes phenomena that occur from a system that is more than the sum of its parts [3]. Feedback describes processes that balance or reinforce other parts of the system [3]. Adaptation describes adjustments to the systems in response to changes [3]. Traditional research that aims to understand or isolate one part of a system, such as considering the impact of a single intervention on individual-level outcomes, may fail to identify the complex impacts of interventions on population health within a complete system [3].

The tools required to conduct systems-level research differ from those traditionally employed in epidemiology and healthcare research. The burgeoning fields of mathematical and simulation modelling have created tools that can be used to represent the complexity of real-world systems and the potential impact of interventions on multiple areas of a population or health care system [4, 5]. System dynamics (SD) models are one type of simulation model that simulate the movement of elements (e.g. people, resources) through a system, controlling for the speed at which elements move through the system as well as other constraints, such as interventions and environmental factors [2]. Notably, SD models incorporate elements of complex systems such as feedback, nonlinearity, and time delays [6].

SD models are highly useful to policy and decision making as they provide a method to examine the impact of polices and interventions on population health and health care using relatively few resources and involving little risk [2]. These models can help identify elements of the system that are sensitive to change as well as factors that can impede or promote the success of initiatives and their potential unintended consequences [2]. Importantly, SD modelling can produce visual representations that simulate alternative policy options to communicate their impacts to decision-makers and those responsible for implementing initiatives and changes [2]. A disadvantage of SD models is that they work less well to capture individual behaviour through the system, which may be better simulated using other types of modeling, such as agent-based modelling [7]. However, they nonetheless provide an innovative tool to examine population-level behaviour.

The value of SD modelling has been acknowledged in public health and SD modelling methods have been developed and applied in the field of infectious and chronic diseases, as well as during health emergencies such as during the COVID-19 pandemic [3, 8]. SD models are also beginning to be applied to mental health [9]. For instance, recent work in Australia has applied SD modelling to examine the impacts of different policy and program approaches on suicide prevention [10, 11]. A recent review identified fourteen articles that used SD modelling in opioid research to understand the complexity and assess the impacts of potential actions and predict future trends [12]. Another area of health that may benefit from an SD modelling approach is depression at the population level. Depression is characterized by feelings of depressed mood, diminished interest, changes in appetite, weight, or sleep, increased fatigue, decreased concentration or decisiveness, and may be accompanied by thoughts of death or suicide [13]. Surveys estimate that 10–25% of people will experience depression in their lifetime and recurrence rates remain high, indicating the need for large-scale interventions [14, 15]. Individual- and social-level factors driving depression include but are not limited to demographic characteristics, socioeconomic status, health behaviours, stressful life events, personal and family history of mental and substance use disorders, mental and physical comorbidities, and community and cultural contexts [16, 17]. Most research on depression tends to examine predictors of depression at the individual level or examines a single isolated part of the system, such as modifying the built environment or reducing financial insecurity [18]. However, it is increasingly clear that population-level depression is characterized by dynamic processes that are resistant to interventions that address only one part of the system [18, 19]. System-level factors driving depression may include screening and diagnosis of depression, access to mental health care, and population-wide health, social, economic and environmental interventions and policies [18, 20]. Conventional research and statistical methods are generally unable to capture the complexity of depression and elements such as dynamic interactions, feedback loops, time delays, and contextual effects. Thus, SD models are needed to account for these elements and provide a system-level perspective of depression.

SD models may also provide a novel perspective on depression by simulating different interventions and scenarios to inform policy and decision-making. Unlike inferential statistical analysis traditionally undertaken in the field of epidemiology, SD models can be applied to assess the potential impacts of a range of different interventions to guide planning and actioning of mental health resources and policies. SD models can also provide a useful tool to examine the potential impact of interventions on multiple aspects of population health and the healthcare system.

However, there is limited information on how SD models have been applied to depression and used to understand the impact of interventions and policies on the population burden of depression. Notably, there is no common framework for the application of SD models to depression or mental health to guide development in this field. In contrast, in the field of infectious disease, the susceptible—exposed—infected—removed (SEIR) model is widely applied and facilitated a rapid public health response to the emerging COVID-19 pandemic. We therefore undertook a scoping review of population-based simulation models of depression that employed SD modelling. While previous reviews have examined the use of simulation models across multiple mental health and substance use outcomes [9, 21], none have focused specifically on depression. Through this review, we aim to generate evidence of the potential for SD models to contribute to understanding the dynamics of depression and the potential impact of interventions. This work provides a foundation of successful strategies and past lessons that can facilitate future work in this emerging area of research. The objective of this review is to identify existing system dynamic models of depression in the general adult population and report on model objectives, elements of SD models, system-level interventions, and quality of reporting that are represented in the literature.

## Methods

We followed the scoping review strategy from the Joanna Briggs Institute Manual for Evidence Synthesis [22].

### Research questions

We defined out main research question as: “What are the characteristics of previous system dynamic models of depression in the general adult population?”.

We defined our sub-questions as:

1. What were the purposes of these models?
2. What were the main elements of system dynamics found in these models related to depression, including definitions of depression, states of depression, transitions between states (i.e. flows), and data collection and calibration?
3. What were the results of these models? What was the impact of potential interventions or policy changes on depression?
4. Did these models report sufficient information to replicate them, according to the STRESS guidelines [23]?

### Search strategy

We followed the PRISMA extension for Scoping Reviews Checklist [24] (Additional file 1: Appendix 2) and guidelines from the Joanna Briggs Institute Manual for Evidence Synthesis for Scoping Reviews [25]. We developed search strategies for peer-reviewed and grey literature that included (1) concepts for depression, major depressive disorders, and dysthymia and (2) concepts for systems thinking, dynamic models, compartmental models, or mathematical models. All search strategies were developed in consultation with a librarian at the Public Health Agency of Canada.

#### Electronic databases

We searched the following databases for peer-reviewed literature: MEDLINE, EMBASE, PsychInfo, and Scopus (inception to October 20, 2021). We searched for the two concepts above in the title, abstract, or keywords. Complete search strategies are provided in Additional file 1: Appendix 1.

#### Grey literature

To include the most recent literature, we also searched MedRxiv pre-print servers (inception to October 20, 2021) and abstracts of conference proceedings from the System Dynamics Society (1983 to 2022).

#### Hand-searching

We searched the references of the two reviews of simulation models of mental healthcare [9, 21] and hand-searched the reference lists of all included articles.

Following our search, all references were exported to Covidence software (covidence.org) and duplicates were removed. The review protocol was not registered on PROSPERO as scoping reviews are currently ineligible for registration in the database.

### Selection criteria and selection process

Titles and abstracts of the records were screened by two independent reviewers. We included studies that presented a SD model of major depressive disorder or similar measures of depression (e.g. elevated depressive systems) in adult populations (age 18 or older) or general populations including all age groups. We excluded articles that presented models of depression or depressive symptoms at the individual level (e.g. changes in depressive symptoms within individuals); other types of simulation models (e.g. agent-based models, discrete-event models); modelled mental health disorders other than depression; modelled depression in paediatric or adolescent populations; and studies that did not present original research (e.g. reviews, commentaries). We excluded conference proceedings and abstracts in languages other than English or French.

### Data charting and quality of reporting assessment

We extracted the following information to answer our research question and sub-questions: model purpose, population, definition of depression, depression states, transitions between depression states (i.e. flows), feedback loops, data used within the model and for calibration, results, potential impact of interventions or policy changes, and replicability of models. To assess the replicability of models, we used the framework of the Strengthening the Reporting of Empirical Simulation Studies (STRESS) guidelines, a standardised checklist for assessing the reporting and replicability of system dynamics models [23]. This framework includes six principles that indicate whether a model reports sufficient information to be replicable: stating the objectives of the study, explaining the logic of the model by providing details to reproduce the results of the base run of the model and any simulation experiments, providing information on data sources and parameters, documenting all software and hardware-specific implementation, and providing the code and information needed to replicate the simulation [23]. One reviewer extracted information which was critically reviewed by another reviewer. Following data extraction, results were compiled in tables and summarized narratively.

## Results

**Fig. 1:**
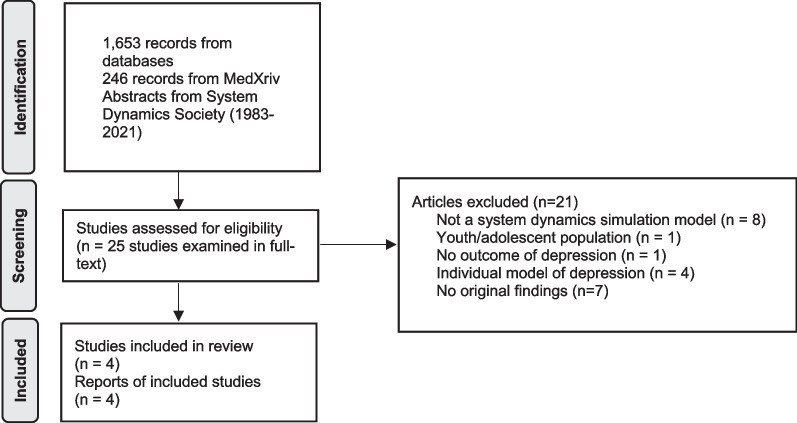
Flow diagram

Figure 1 presents the flow chart of our study selection. We identified 559 citations from MEDLINE, 533 from Embase, 391 from PsychInfo, 733 from Scopus, and 420 from MedXRiv. After deleting repeated citations, we screened titles and abstracts from 1899 citations from these sources. We also screened titles and abstracts from the System Dynamics Society from 1983 to 2022. After title and abstract screening, we carried 25 citations forward to full-text screening. Four studies were included in the final review. Reasons for exclusion were not using SD models (*n* = 8), population other than adults or the general population (*n* = 1), outcome other than depression (*n* = 1), individual-level models of depression (*n* = 4), and publication was not primary research (*n* = 7).

**Fig. 2:**
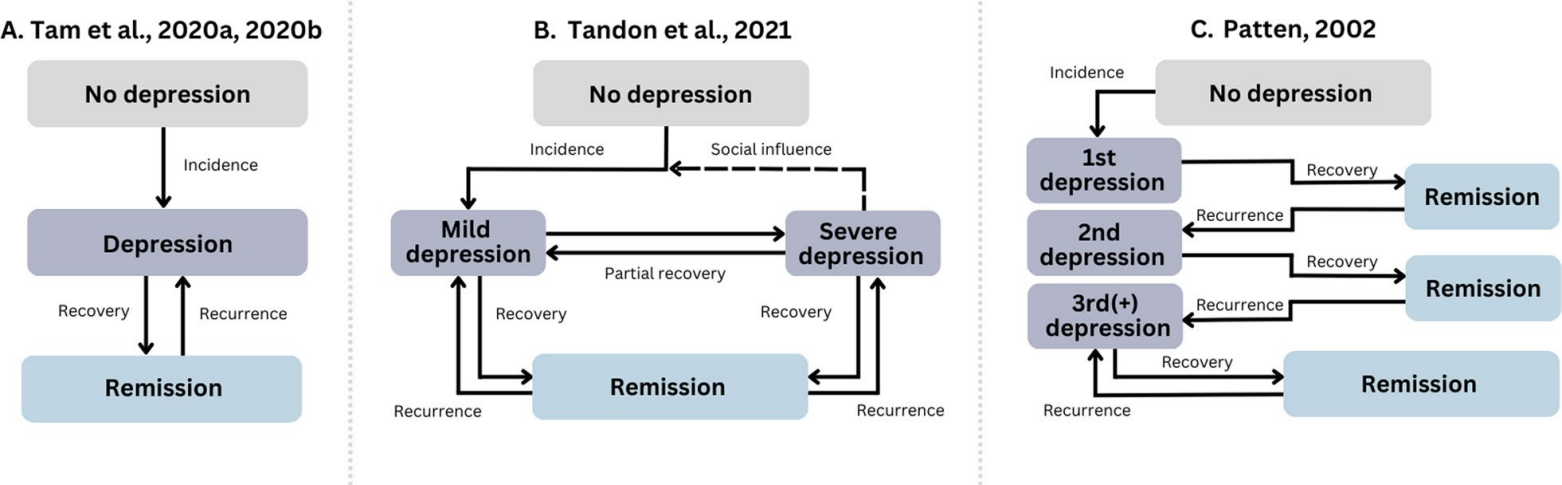
Schematic representation of depression states and flows in Tam et al. [27, 28] (**A**), Tandon et al. [29] (**B**), and Patten [26] (**C**). The schematics exclude other parts of the system dynamics models for illustrative purposes. See Table 1 for details on study characteristics and modelling strategies

We extracted information from four studies, with characteristics presented in Table 1 and schematic representations in Fig. 2. These models were published between 2002 and 2021 and were applied in Canada (*n* = 1), the USA (*n* = 2), and Zimbabwe (*n* = 1). For our first sub-question, we assessed the purpose of these models. While all models aimed to assess the impact of system elements or clinical / public health interventions on depression, interventions varied widely. The first model was published in 2002 by Patten with the purpose of describing the dynamics of major depressive episodes in the general Canadian population, examining the impact of increased antidepressant treatment and long-term preventive treatment [26]. In 2020, Tam and colleagues published two models of depression in the United States [27, 28]. The first model aimed to assess the impact of recall error on USA lifetime estimates of depression [27]. The second model built on the first and estimated smoking prevalence, smoking-attributable mortality, and life-years lost due to smoking among adults with and without depression [28]. This model further aimed to estimate the impact of smoking cessation and no new smoking initiation among adults with depression on depression estimates [28]. Lastly, Tandon et al. developed a model in 2021 with the purpose of estimating the impact of increasing incidence rates of depression and increasing counselling rates on depression in the population of Zimbabwe [29].

**Table 1:** Study characteristics and modelling strategies

	Patten 2002 [26]	Tam 2020a [27]	Tam 2020b [28]	Tandon 2021 [29]
Purpose	Evaluate the impact of antidepressant medication use and long-term preventive treatment on the prevalence of MDE	Evaluate the impact of recall error on the prevalence of lifetime MDE	Evaluate trends in smoking, smoking-attributable mortality, and life-years lost by MDE status. Evaluate the impact of smoking cessation among adults with depression	Estimate the impact of counselling rates and transmissibility of depression through social connections on depression estimates
Population	Canada general population	USA general population	USA population aged 18 +	Zimbabwe general population
Definition of depression	Past-year MDE using the CIDI-SF. Current MDE as past-year MDE and current psychological distress	Lifetime and past-year MDE using DSM-IV criteria	See Tam 2020a	Current depression defined as an extreme case of negative emotion
Depression states	• Never depressed, first, second, or third/subsequent MDE • First, second, and third episodes of remission • Short duration/good prognosis, intermediate duration/prognosis with antidepressant use, intermediate duration/prognosis without antidepressant use, long duration/poor prognosis	• Never reported MDE • Past-year MDE • Former MDE • Former MDE with recall error	MDE states (see Tam 2020a) among people who have never smoked, people who smoked previously, people who currently smoke	• No depression • Primary depression, mild or initial stage • Secondary depression, severe or acute stage • Recovered depression
Transitions between depression states (i.e. flows)	• Incidence of first MDE • Remission of first, second, and third/subsequent MDE • Recurrence of second and third/subsequent MDE from first, second, or third/subsequent MDE	• Incidence of past year MDE • Recovery from past year MDE to former MDE • Incidence of underreporting from former MDE to recall error • Recurrence of past year MDE from former MDE or recall error	See Tam 2020a among adults who are current, former, or never smokers	• Incidence of primary depression • Recovery of primary depression • Incidence from primary to secondary depression • Recovery of secondary depression
Feedback loops	• Feedback loop between incidence of third/subsequent MDE and recovery of third/subsequent MDE	• Feedback loop between former MDE and past year MDE • Feedback loop between former MDE, recall error, and past year MDE	See Tam 2020a among different categories of smoking status	• Number of people with secondary depression creates feedback loop by influencing incidence rate of primary depression
Data used for depression and parameter estimates	• National epidemiologic studies (National Population Health Survey, 1996 cycle; Stirling County study) • Literature reviews	• National epidemiologic studies (Epidemiologic Catchment Area Study) • Calibrated estimates	See Tam 2020a	• Unknown sources for fixed parameters • Calibrated estimates
Calibration data	National Population Health Survey 1996 cycle	National Survey on Drug Use and Health 2017, Baltimore ECA study 1981–2005	See Tam 2020a	Annual report of cases of depressed population in Zimbabwe from 1991 to 2017
Interventions or policy changes	Increasing antidepressant use among people with intermediate prognosis of MDE Changes in recurrence rates for people with three or more MDEs though long-term treatment		Maximum potential reduction in premature mortality scenario, where all smokers with MDE quit immediately in 2018 and no new smoking initiation occurs	Increase in rate of counselling among people with primary and secondary depression
Is model replicable	Yes	Yes	Yes	No

*CIDI-SF:* Composite International Diagnostic Interview—Short Form, *MDE*: Major depressive episode

We examined depression states, transitions, applications of data and data calibration, and feedback loops to answer our second sub-question on elements of system dynamics found in these models. In three of these models, depression was defined as a major depressive episode (MDE) according to DSM-IV criteria [26–28]. Patten et al. modeled current MDE, defined as a positive screen for past-year MDE, measured using the Composite International Diagnostic Interview—Short Form (CIDI-SF) and current psychological distress [26]. States or stocks for depression included no depression; first, second, and third/subsequent episodes of depression; and remission after a first, second, or third/subsequent episode [26]. Among each of these stocks, depression was further divided into short duration/good prognosis, intermediate duration/prognosis with treatment, intermediate duration/prognosis without treatment, and long duration/poor prognosis [26]. The recovery rates of depression and the frequency of antidepressant use in each group were estimated using national Canadian survey data [26]. Tam et al. modeled both lifetime and past-year MDE [27, 28] based on self-reported DSM-IV criteria of MDE. Stocks included never having experienced a MDE; past-year MDE; former MDE; and former MDE with recall error (i.e. not reporting former MDE) [27, 28]. Finally, Tandon et al. defined depression as an extreme case of negative emotions, conceptualizing depression as a disorder that spreads socially [29]. Stocks included never having depression; primary or initial stage of depression; secondary or acute depression that can spread socially; and recovered depression [29].

All models included flows for incidence and recurrence of depression, though they were parameterized differently. In Patten’s model, the incidence of a first episode of depression was set to 0 to better reflect age-specific prevalence rates observed in the calibration dataset and in other literature [26]. Recurrence rates were drawn from the literature [26]. Tam et al. applied age-specific incidence rates of first MDE and recurrence rates from a large US survey [27, 28]. They also calibrated age-specific incidence rates for younger people to match nationally-representative prevalence rates of depression [27, 28]. In their model of smoking rates, Tam et al. also calibrated recurrence and recovery rates of depression among different smoking groups [28]. Tandon et al. calibrated the incidence rate of primary and secondary depression to reported cases of depression in Zimbabwe [29]. When examining recovery of depression, Patten and Tam et al. estimated recovery or remittance rates using survey data [26–28], while Tandon et al. estimated recovery from primary depression using calibration techniques and used a fixed value for recovery from secondary depression, though the source of this value was unclear [29].

Feedback loops, a defining characteristics of system dynamics modelling, were present in all models (see Fig. 2). In Patten’s model, there was a feedback loop from third/subsequent depression through remittance to a recovered state, and back to an MDE through recurrence [26]. In the two models presented by Tam et al., there was a feedback loop from past-year MDE to former MDE through recovery and recurrence of MDE, respectively [27, 28]. In Tandon et al., secondary or acute depression had a direct influence on the incidence rate of primary depression, reflecting the conceptualization of depression as a contagious condition [29]. These feedback loops allowed the models to represent the repeated nature of depressive episodes within populations.

Our third sub-question examined the results of these models, especially with respect to the impact of interventions or policy changes on population-level depression. Interventions varied considerably between studies. Patten et al. reported that 2% of all person-time among adults would be in a state of a MDE [26]. Among adults with any depressive episodes, 13% of their lives would be spent in a MDE [26]. An intervention that increased antidepressant use would only have a small impact on the prevalence of depression. On the other hand, reducing the recurrence of multiple depressive episodes through long-term preventive treatment could contribute to meaningfully reduce the prevalence of depression in the Canadian population [26]. In their model focusing on recall error, Tam et al. reported results that 24% of USA adults experience at least one MDE in their lifetime when adjusting for recall error [27]. Approximately 13% of women and 7% of men with past MDEs do not report them due to recall error [27]. This article did not examine interventions or policy changes. In their model of depression and smoking, Tam et al. reported that approximately 484 000 smoking-attributable deaths will occur between 2018 and 2060 among people with MDEs [28]. While smoking rates are expected to decrease to 2060, relative decreases will be smaller among those with MDEs [28]. They also examined the impact of an intervention if no new initiation of smoking occurred and everyone who smoked quit in 2018. This would result in 7.5 million life years being gained and up to 264 000 deaths being prevented by 2060 [28]. Tandon et al. examined the impacts of increasing incidence rates of depression and increasing counselling rates on the depression state equilibrium (i.e., stability of prevalence rates of depression in the population) in Zimbabwe [29]. Increasing incidence rates of primary and secondary depression could lead to a population with a high but stable prevalence of depression. Increasing counselling rates, especially among people with secondary or acute depression, could lead to a population with a stable prevalence rate of no depression [29].

Our final research question examined whether these models were replicable, using the framework of the STRESS guidelines [23]. Results are presented in Table 2 for each of six principles in this framework, with further information on each criterion available in [23]. Firstly, all models met the principle of describing their objectives, including the background, policy analysis, experiment design, and optimization, as applicable. The second principle refers to explaining the logic of these models. While all articles explained the logic of their model in text, only Patten [26] and Tam et al. [27, 28] provided model diagrams. For the third principle, Patten [26] and Tam et al. [27, 28] described all model components in detail. Tandon et al. [29] did not fully describe their model flows or sources. The fourth principle includes describing data sources, data processing, input parameters, and assumptions. This was fulfilled in Patten [26] and Tam et al. [27, 28]. Tandon et al. [29] did not describe their data sources or assumptions in detail. Fifthly, none of the models fully described their implementation, as they did not specify the hardware or model time. Finally, only Patten [26] and Tam et al. [27, 28] provided information on simulation software and/or code to replicate the model. Overall, three of these models [26–28] were considered replicable.

**Table 2:** Quality of Study Reporting using the Strengthening the Reporting of Empirical Simulation Studies (STRESS) guidelines—System Dynamics Guidelines (STRESS-SD)

Section, item, and recommendation	Patten 2002 [26]	Tam 2020a [27]	Tam 2020b [28]	Tandon 2021 [29]
**1. Objectives**				
1.1 Explain the background and rationale for the model	Yes	Yes	Yes	Yes
1.2 Describe all outcome variables that are reported. Include details on how they are calculated during the model run	Yes	Yes	Yes	Somewhat. Does not specify numeric output of stocks in the model. Unclear which stocks are related to outcome
1.3 If the model has been used for policy analysis (user-designed experiments) and policy formulation (multiple experiments to obtain best policy), state the research questions that it was used to answer	Yes	NA	Yes	Yes
1.3 a.) Policy based analysis—Provide a name and description of each policy tested, providing a rationale for the choice of policies and parameters employed	Yes	NA	Yes	Yes
1.3 b.) Design of experiments—Provide details of the design and the parameters that will be used	Yes	NA	Yes	Yes
1.3 c.) Simulation Optimisation—Provide full details of what is to be optimised and the parameters that will be included and the algorithm that will be used	NA	NA	Yes	Yes
**2. Logic**				
2.1 Base model overview diagram. Provide one or more causal loop, stock and flow (a.k.a level and rate) or equivalent diagrams to describe the basic logic of base model to readers	Yes	Yes	Yes	No
2.2 Base model logic. Give details of the base model logic in terms of feedback loops	Yes	Yes	Yes	Yes
2.3 Scenario logic. Give details of the logical difference between the base case model and policies, scenarios and experiments	Yes	NA	Yes	Yes
2.4 Algorithms. Provide detail on any algorithms, functions or equations that mimic complex or manual processes in the real world	Yes	Yes	Yes	NA
2.5 Components				
2.5.1 Stocks/Levels. Give details of all stocks within the simulation including a description of their role in the model	Yes	Yes	Yes	Yes
2.5.2 Flows/Rates. List all flows within the model along with units and equations. Describe the role of flows in the model e.g. if they have a delay	Yes	Yes	Yes	Somewhat. Does not include units
2.5.3 Constants/Converters/Auxilliary. List all variables within the model and detail their equations (if applicable) including units	Yes	Yes	Yes	NA
2.5.4 Graphical Functions / lookup tables. List and detail all graphical functions within the model and describe their data sources	NA	NA	NA	NA
2.5.5 Sources / sinks. Give details of the model boundaries i.e. all infinite sources and sinks within the model	Yes	Yes	Yes	No
**3. Data**				
3.1 Data sources. List and detail all data sources	Yes	Yes	Yes	No, does not detail data sources
3.2 Pre-processing. Provide details of any data manipulation that has taken place before its use in the simulation	Yes	NA	NA	NA
3.3 Input parameters. List of all input variables in the model, provide a description of its use and include parameter values	Yes	Yes	Yes	Yes
3.4 Where data or knowledge of the real system is unavailable what assumptions are included in the model?	Yes	Yes	Yes	Unclear
**4. Experimentation**				
4.1 Initialization. List all initial stocks and auxiliary variables within the model	Yes	Yes	Yes	No
4.2 Run length. Describe the run length of the simulation model and time units	Yes	Yes	Yes	Unclear
4.3 Estimation approach	Yes	Yes	Yes	Yes
**5. Implementation**				
5.1 Software or programming language. State the operating system and version and build number	NA	Yes	Yes	No
5.2 Random sampling. State the algorithm used to generate random samples with in the software/programming language used	NA	Yes	Yes	NA
5.3 Model execution. Report the integration method used along with time step settings	Yes	Yes	Yes	No
5.4 System specification. State the model run time and specification of hardware used	No	No	No	No
**6. Code access**				
6.1 Computer model sharing statement. Describe how someone could obtain the model described in the paper, the simulation software and any other associated software (or hardware) needed to reproduce the results	Yes	Yes	Yes	No

## Discussion

The main research question of this scoping review aimed to identify characteristics of existing system dynamics models of depression in the general adult population. Our objective was to identify the potential for SD models to contribute to understanding the dynamics of depression within populations and simulate possible impacts of interventions or policy changes. We identified four system dynamics models of depression that demonstrate the application of SD modelling to depression research. All four models applied SD methods to examine population-level depression and incorporated elements unique to systems modelling, such as feedback loops and calibration of unknown data. Three models also examined the potential role of a variety of interventions on population-level mental health and other outcomes, demonstrating their utility for policy and decision-making.

This review adds to the prior literature demonstrating the potential of SD modelling within the field of population mental health. Two reviews have examined simulation modelling in a mental health context, but did not identify any of the models of this review [9, 21]. Firstly, in a review of 160 studies, Long and Meadows reported an increase in the use of simulation modelling in mental healthcare in the last two decades, but SD modelling was rarely used and mostly applied to system flows (such as patient flow and resources optimization) [9]. Secondly, these findings were reflected in a more recent review of 253 studies that examined the application of SD modelling to healthcare, and also found that SD was rarely used in population-level mental health [21] Our review contributes to this growing body of research by identifying and synthesizing how SD models have been applied to depression. As depressive disorders are the most common mental illnesses worldwide [30], applying SD models to understand depression at the population level and to visualize the potential of clinical and public health interventions may have considerable impact.

Through our research sub-questions, we compared the following among the four models identified: model purpose, system dynamics elements (i.e. depression states, transitions between states, feedback loops, data application and calibration), model results and the impact of interventions, and whether each model reported sufficient information to be replicable. Notably, the identified studies modelled depression differently, including diverse stocks and flows for depression severity, recurrence, remittance, and recall error, and applied their models to a range of different health interventions. However, they all integrated SD elements such as flows for incidence and recurrence of depression, and feedback loops. Through our assessment, we identified several lessons learned and future directions for research and public health.

The models included in this review demonstrated the usefulness of applying SD models to incorporate the complexity of depression as a population-level illness. Notably, all four models presented different approaches to modelling depressive illness. Patten et al. considered depressive episodes of different durations as well as distinct parameters for incidence and recovery [26]. Tandon et al. included two stages of depression, a primary or initial stage and an acute or severe stage [29]. Furthermore, models by Patten and Tam et al. included feedback loops for recurrence of MDE, allowing members of the population to experience more than one MDE throughout their lifetime [26–28]. Taking another approach, Tandon et al. included a feedback loop such that the number of people with acute depression impacted the incidence of primary depression on the assumption that depression can spread in a population [29]. The models presented in this review underscore the utility of applying SD modeling techniques to represent complex characteristics of depression, such as depression severity or duration of episodes, recurrence, and recovery. Models developed by Tam et al. further point to the need for modeling age- and sex-specific depression dynamics [27, 28]. Notably, despite these advances, all models remained relatively simple and none combined all aspects of depression represented in the literature. In their review on simulation modeling in mental health, Long et al. noted the challenges of capturing the complex biopsychosocial interactions related to individual illness (e.g. sex, age), severity or symptom profiles, treatment, and social and environmental factors [9]. Future models may consider integrating additional or more detailed aspects of depressive illness, such as including both duration and severity of depression.

The results of this review also demonstrate the utility of SD modeling to develop plausible parameter estimates when limited evidence is available using methods such as calibration. These methods would not be available using conventional epidemiologic study design and statistical methods. Patten’s model initially implemented incidence rates of MDE from survey-based estimates, but was revised to match age-specific prevalence rates [26]. Tam et al. calibrated their model using survey-based estimates to develop parameters for recall error for MDEs as well as age-specific incidence rates of MDEs for some of the population [27]. In their model that included smoking, Tam et al. used literature estimates as a starting point and further implemented an optimization algorithm to specify upper and lower limits for several unknown parameters [28]. These parameters included MDE incidence for younger populations, probability of incidence of MDE among current and former smokers, MDE recurrence among smokers, and MDE recovery rates among current and former smokers [28]. Tandon et al. developed initial values of incidence rates of depression using Latin hypercube sampling techniques and estimated parameters values using Markov Chain Monte Carlo, calibrating to observed rates of depression [29]. All studies included in this review needed to estimate unknown parameters, indicating the need for methods such as SD modelling that can incorporate these techniques.

On the other hand, these findings also indicate the need for improved population-level information on mental health characteristics to be used in model parameters and calibration. In the models presented here, depression parameters were often obtained from national or regional surveys such as the National Population Health Survey in Canada [26] and the Epidemiologic Catchment Area Study in the USA [27, 28]. However, there are limitations in using survey data. Many population-based surveys, such as the National Population Health Survey[26], use screening tools for depression, which may overestimate the prevalence of depression. As well, Tam et al.’s model demonstrated that prevalence estimates may also underestimate lifetime estimates of depression due to recall error, particularly among older adults [27]. Notably, all models needed to use other sources of information, such as literature reviews and calibration methods, as estimates available from primary data did not correspond to the required parameters. Population-level estimates of a variety of aspects of depression, such as incidence, recurrence, and recovery, are needed to develop accurate models, including changes in parameters over time [31].

The models presented here also show the usefulness of using SD models to estimate the impact of large-scale public health interventions on the prevalence of depression. In Patten’s model, which was applied to the Canadian population, increasing antidepressant use had only a limited impact on the prevalence of MDEs compared with the larger impact of decreasing recurrence rates of MDE among those with chronic depression [26]. Furthermore, Patten provided a visual to present the results of their interventions, facilitating its application to policy-making. Tandon et al. found that increasing counselling rates for people with primary and secondary depression may eventually lead to a population with nearly no depression [29], although such scenario remains theoretical and may not be realistic. Thus, these models demonstrate the utility of SD modelling to decision-making and policy-making, allowing for comparisons of different interventions in a simulated environment. Nonetheless, there remain many interventions that should be explored, such as prevention of initial depressive episodes and age- and sex-specific interventions. Importantly, some interventions may be more effective for specific types of depressive episodes or in specific populations. For instance, psychotherapy may be more effective in younger adults compared to older adults [32] and sex differences in antidepressant efficacy may exist [33]. While these results present preliminary evidence that SD models of depression may be an innovative tool to inform decision-making, this field has the potential for further development and complexity.

Finally, the results of this review underpin the need for high-quality, reproducible reporting of modelling studies. Three of the four studies were considered replicable, but all studies had room for improvement in reporting. Strong reporting of models will facilitate knowledge sharing and increase transparency for planning and decision-making, using checklists such as the Strengthening the Reporting of Empirical Simulation Studies [23]. Modelling could be incorporated into accessible tools that could inform stakeholders on the potential impacts of interventions.

This review provides the first in-depth synthesis of the SD models that have been applied to depression at the population level and has several strengths. We examined nearly 1900 titles and abstracts as well as searching grey literature to identify SD models of depression. We presented a detailed summary of each model’s purpose, SD modelling elements (i.e. depression states, transitions, feedback loops, data), results and interventions, and replicability. We synthesized this information, demonstrating the utility of SD modelling for population-level depression as well as the potential to inform policy and decision-making by presenting results for interventions and policy changes. We also identified lessons learned and areas of future research.

This review also has several limitations. Although we searched several databases as well as pre-print servers, some articles may have been missed. Our scoping review was also limited to English and French articles. We did not assess the risk of bias of the study parameters as this was beyond the scope of our objectives. We focused on SD models but recognize that other simulation models may also be appropriate to study population-level depression (see Long and Meadows [9] for a review of other simulation approaches).

## Conclusions

While used extensively to model infectious and some chronic diseases, SD modelling is also an emergent and useful tool to examine the complexity of population-level dynamics of mental health outcomes, including depression. This scoping review identified four system dynamics models of depression among adult populations. Though SD modelling has not been widely applied in the area of mental health, these models highlight the complexity of modeling depression and the need for techniques beyond conventional epidemiologic methods. From a public health perspective, these SD models highlight the applicability of modelling to understand the dynamics of depression as well as the potential impact of interventions. These models presented findings on the impact of a variety of interventions on population-level depression, demonstrating their utility in policymaking and decision making on a large scale. While SD models are not yet widely applied to depression, they show promise in modelling the complexity of mental health and health systems as well as a unique policy tool for assessing the potential impact of interventions.

## Supplementary Information

**Additional file 1.** Appendices 1 & 2.

## Data Availability

Not applicable.

## Abbreviations

MDE: Major depressive episode
SD: System dynamics
STRESS: Strengthening the Reporting of Empirical Simulation Studies
